# Comparing Diagnostic Coding and Laboratory Results

**DOI:** 10.3201/eid1107.041058

**Published:** 2005-07

**Authors:** Asha J. Riegodedios, Anuli Ajene, Mark A. Malakooti, Joel C. Gaydos, Victor H. MacIntosh, Bruce K. Bohnker

**Affiliations:** * Navy Environmental Health Center, Portsmouth, Virginia, USA;; † Department of Defense Global Emerging Infections Surveillance and Response System, Silver Spring, Maryland, USA

**Keywords:** public health surveillance, international classification of diseases, evaluation studies

**To the Editor:** The global Military Health System maintains electronic inpatient (Standard Inpatient Data Record, SIDR) and outpatient (Standard Ambulatory Data Record, SADR) clinical diagnostic coded data generated by the Department of Defense Composite Health Care System (CHCS), an electronic system that tracks and stores administrative and other patient encounter data. Because these records are readily available, widespread monitoring of these data as a means of medical surveillance has been suggested (1,2). Only 1 study in the literature assessed electronic coding reliability of these data (3); those authors found SIDRs to be a reliable source of billing data for common diagnoses, not including notifiable infectious diseases. We compared SADR and SIDR infectious disease diagnostic codes to laboratory data to assess the usefulness of these datasets in notifiable disease surveillance.

We identified SADRs and SIDRs coded for malaria, syphilis, acute hepatitis B, and Lyme disease in sailors, marines, and their family members, who were beneficiaries for medical care in a large metropolitan area. Medical encounters from January 1, 2001, to June 30, 2002, were studied. All records for the same patient with the same diagnostic code(s) were considered as 1 encounter. Records were selected on the basis of International Classification of Diseases, Ninth Revision, Clinical Modification (ICD-9-CM) codes (4) as defined by the Department of Defense (5). Laboratory data were not part of SIDRs and SADRs but were part of CHCS.

For records with diagnostic codes relating to any of the 4 diseases of interest, laboratory records were searched to determine: 1) whether the provider ordered an appropriate test or tests and 2) if these were ordered, were the test results confirmatory (positive). Appropriate and confirmatory test results were determined by using published references (5–7) and local laboratory practices. For malaria, a blood smear was considered an appropriate test with a positive blood smear accepted as confirmatory (5,6). We considered both nontreponemal and treponemal tests to be appropriate for syphilis but only a positive treponemal test as confirmatory (5,6). For acute hepatitis B, we considered hepatitis B surface antigen or immunoglobulin (Ig) M anti-hepatitis B core (anti-HBc) to be an appropriate test, but only a positive IgM anti-HBc was accepted as confirmatory (5,6). We considered enzyme immunoassay total antibody screens or Western blot (WB) IgG or IgM tests to be appropriate for Lyme disease and accepted any positive test as confirmatory (5–7). χ^2^ calculations were conducted (α = 0.05).

Twenty-one SIDRs and 155 SADRs met the selection criteria (Table). While 61.9% of SIDRs studied had appropriate laboratory tests ordered, only 19.0% had associated confirmatory results in CHCS. For outpatient records, 64.5% had appropriate tests ordered, and 15.5% had confirmatory results. Among the SADRs, the proportions of appropriate laboratory tests for the diseases studied differed significantly (summary χ^2^ =11.5, p = 0.01). These results suggest that tracking electronic SADR and SIDR datasets for the selected reportable diseases could produce a high number of false-positive reports; in this study, 81.0% of inpatient and 84.5% of outpatient reports would lack a confirmatory laboratory test result.

**Table:** Clinical records with associated laboratory test results*

Disease†	Inpatient records (SIDR)			Outpatient records (SADR)		
	No. records selected	No. tests ordered (%)	No. confirmatory results (%)‡	No. records selected	No. tests ordered (%)§	No. confirmatory results (%)‡¶
Malaria	3	3 (100.0)	1 (33.3)	17	8 (47.1)	1 (5.9)
Syphilis	1	1 (100.0)	1 (100.0)	44	31 (70.4)	12 (27.3)
Acute hepatitis B	16	8 (50.0)	1 (6.3)	39	32 (82.1)	5 (12.8)
Lyme disease	1	1 (100.0)	1 (100.0)	55	29 (52.7)	6 (10.9)
Total	21	13 (61.9)	4 (19.0)	155	100 (64.5)	24 (15.5)

*This table presents Standard Inpatient Data Records (SIDR) and Standard Ambulatory Data Records (SADR) studied and percentages with appropriate laboratory tests ordered and confirmatory laboratory test results. †International Classification of Diseases, 9th Revision, Clinical Modification (ICD-9-CM) codes: malaria, 084.0–084.6; syphilis, 090, 091, 095, 096; acute hepatitis B, 070.30, 070.31; Lyme disease, 088.81. ‡Percentages reported reflect the proportion of records that had positive confirmatory laboratory results. §Summary χ^2^ = 11.5; p = 0.01. ¶Summary χ^2^= 7.0; p = 0.07.

This initial evaluation is limited but supports the need to evaluate electronic datasets before using them for medical surveillance. We examined only ICD-9-CM coded records of selected diseases from 1 geographic area, with resulting small samples. Therefore, our results may not be generalizable. This study was restricted to laboratory, inpatient, and outpatient data recorded within 1 coordinated military system. Laboratory testing or clinical visits may have occurred outside of this network and may not have been captured in this study. Laboratory data were not recorded or stored in a standardized format in CHCS, increasing the likelihood of misclassification. We did not evaluate all related sources of data, including the hard-copy clinical records, so we do not know the completeness of the ICD-9-CM codes or the extent of ICD-9-CM code misclassification. Additionally, local clinical practices in terms of both ordering laboratory tests and coding diagnoses for the diseases studied were not defined.

Future studies would benefit from comparing reported medical events, paper medical records, and electronic datasets to include determination of sensitivity as well as positive predictive value (2,8,9). Discordance in these data sources should be investigated for miscoding, incomplete data, and unexpected clinical practices.

Efforts to improve medical record coding at military medical treatment facilities are under way (10). Additionally, standardization of CHCS laboratory test files, including adoption of the Logical Observation Identifiers Names and Codes system for standardized reporting of test names, is under way (available from http://www.ha.osd.mil/policies/2003/03-023.pdf). However, a documented, complete, reliable, and closely monitored single source of data for medical surveillance and disease reporting does not currently exist. Therefore, surveillance programs for infectious diseases in the US military should include monitoring of multiple, related sources of data and information (e.g., electronic inpatient and outpatient encounters, laboratory results, and pharmacy data). All of these sources should be evaluated for completeness and accuracy.
